# *In Vitro and In Silico* Risk Assessment in Acquired Long QT Syndrome: The Devil Is in the Details

**DOI:** 10.3389/fphys.2017.00934

**Published:** 2017-11-16

**Authors:** William Lee, Monique J. Windley, Jamie I. Vandenberg, Adam P. Hill

**Affiliations:** ^1^Molecular Cardiology and Biophysics Division, Victor Chang Cardiac Research Institute, Darlinghurst, NSW, Australia; ^2^St. Vincent's Clinical School, University of New South Wales, Sydney, NSW, Australia

**Keywords:** kv11.1, herg, acquired long QT syndrome, arrhythmia, pharmacology, CiPA, modeling

## Abstract

Acquired long QT syndrome, mostly as a result of drug block of the Kv11. 1 potassium channel in the heart, is characterized by delayed cardiac myocyte repolarization, prolongation of the T interval on the ECG, syncope and sudden cardiac death due to the polymorphic ventricular arrhythmia Torsade de Pointes (TdP). In recent years, efforts are underway through the Comprehensive *in vitro* proarrhythmic assay (CiPA) initiative, to develop better tests for this drug induced arrhythmia based in part on *in silico* simulations of pharmacological disruption of repolarization. However, drug binding to Kv11.1 is more complex than a simple binary molecular reaction, meaning simple steady state measures of potency are poor surrogates for risk. As a result, there is a plethora of mechanistic detail describing the drug/Kv11.1 interaction—such as drug binding kinetics, state preference, temperature dependence and trapping—that needs to be considered when developing *in silico* models for risk prediction. In addition to this, other factors, such as multichannel pharmacological profile and the nature of the ventricular cell models used in simulations also need to be considered in the search for the optimum *in silico* approach. Here we consider how much of mechanistic detail needs to be included for *in silico* models to accurately predict risk and further, how much of this detail can be retrieved from protocols that are practical to implement in high throughout screens as part of next generation of preclinical *in silico* drug screening approaches?

## Introduction

In the past 20 years, a range of structurally unrelated drugs, including antihistamines, antibiotics and antipsychotics, have been withdrawn from the market due to adverse effects on cardiac repolarization - so called acquired long QT syndrome (aLQTS). aLQTS is characterized by prolongation and sometimes morphological deformation of QT segments on the 12-lead electrocardiogram (ECG), syncope and sudden cardiac death due to the polymorphic ventricular arrhythmia Torsade de Pointes (TdP). Theoretically, aLQTS can occur due to unwanted drug induced modulation of any of the ionic channels that contribute to cardiac repolarization either through direct modulation of channel conductance (Cavero et al., 2000; Perrin et al., 2008a) or up/down regulation of channel trafficking and expression on the cell membrane (Dennis et al., 2007; Ballou et al., 2015). In practice however, the overwhelming majority of these drugs cause aLQTS through blockade of the Kv11.1 potassium channel that carries the rapid component of the delayed rectifier current in the heart (I_Kr_) (Perrin et al., 2008b).

As a result of the prevalence of these proarrhythmic side effects, regulatory guidelines have been put in place as part of preclinical drug development to ensure such dangerous compounds do not get to market. In their current form, these guidelines use simple steady-state measures of Kv11.1 inhibitory concentration and action potential prolongation to estimate arrhythmic risk (E14, 2005; S7B, 2005). However, while these steady-state measures of Kv11.1 block are very sensitive (no new proarrhythmic drugs have knowingly come to market since the inception of these guidelines), they are not specific. The link between Kv11.1 block, repolarization delay, and TdP is poorly understood meaning these measures are poor surrogates for actual risk of TdP. Given that not all drugs that block Kv11.1 are going to be proarrhythmic, this has likely resulted in an unnecessarily high attrition rate of drugs in development (Redfern et al., 2003; Sager et al., 2014).

**Figure 1:**
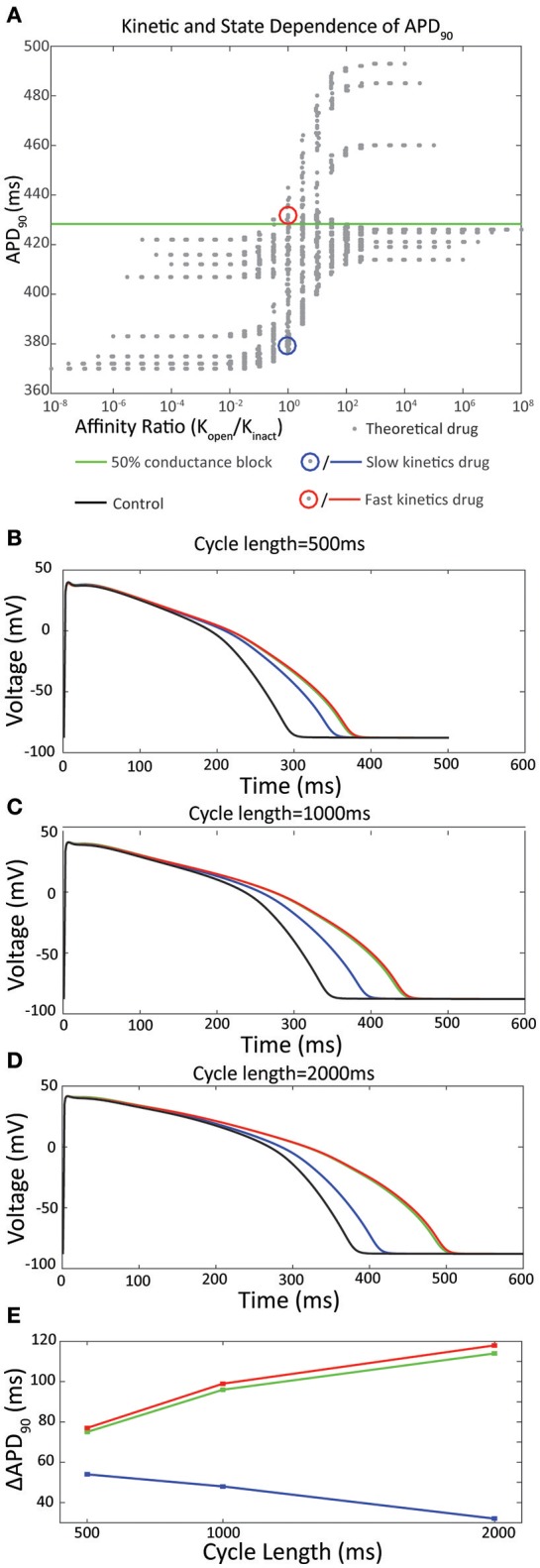
**(A)** *In silico* analysis of APD_90_ with respect to the ratio of affinity for the open vs inactivated state, *K*_o_ /*K*_i_. A family of theoretical drugs was constructed using permutations of the forward and reverse rates for binding to the open state of the channel (*k*f,open and *k*b,open respectively) and the inactivated state of the channel (*k*f,inact and *k*b,inact respectively) in the range 0.01–100 s-1 using half-logarithmic increments. An IC_50_ dose of each drug, calculated *in silico* using a simulated direct drug application of the drug at a holding potential of 0 mV, was applied to the O'Hara Rudy action potential at 1000 ms pacing cycle length. Drugs with higher affinity for the open state are shown on the right (*K*_o_ / *K*_i_ > 1) and drugs with higher affinity for the inactivated state are shown to the left (*K*_o_ / *K*_i_ < 1). The green line shows the APD_90_ for IK_r_50 (a 50 % conductance block of I_kr_) of 428 ms. *Adapted from* Lee et al. (2016). **(B–E)** Drug binding kinetics contribute to reverse rate dependence. **(B)** Action potentials simulated at 500 ms pacing interval in response to an IC_50_ dose of the drugs selected in **(A)**. Black line represents a control action potential with no drug applied. **(C)** Action potentials at 1,000 ms pacing interval. **(D)** Action potentials at 2,000 ms pacing interval. **(E)** Pacing cycle dependence of ΔAPD_90_.

To address this issue, the Comprehensive *in vitro* Proarrhythmia assay (CiPA) has been proposed as a new safety paradigm in understanding TdP and assessing proarrhythmia risk (Sager et al., 2014). CiPA has two primary objectives: (1) Detailed *in vitro* electrophysiological characterization of drug interaction with Kv11.1 (and other cardiac ion channels) and integration of this data into *in silico* models to predict proarrhythmia in simulations of the cardiac action potential and (2) Validation of *in silico* models using human induced pluripotent stem cell derived cardiac cardiomyocytes (Fermini et al., 2016). Central to the first of these objectives is our understanding of the mechanistic subtleties of how drugs interact with Kv11.1. There are several key factors that contribute to a drug's pharmacological profile and hence contribute to proarrhythmic risk, including the kinetics of drug binding and unbinding, gating-state preference and temperature dependence. These factors are not easily quantified by simple steady-state measures, yet can have significant effects on the measured potency of a drug as well as profound impact on the degree of repolarization delay and the emergence of proarrhythmic markers seen in *in silico* simulations. For example, Figure 1 demonstrates an *in silico* simulation of 6,561 theoretical drugs that block Kv11.1 all at calculated IC_50_ doses and yet the simulated action potential prolongation is significantly varied. Furthermore, whilst Kv11.1 is the major repolarizing current in the cardiac action potential, there are multiple other currents that contribute to repolarization, a concept known as repolarization reserve (Roden, 1998). In this context, the evolution of drug induced TdP may involve block of multiple ion channel currents and a drug's affinity for a variety of targets may modify the proarrhythmic risk associated with its block of Kv11.1. Determination of the proarrhythmic risk profile of Kv11.1 blocking drugs is therefore a multifaceted problem that goes beyond simple measures of potency. As a result, using *in silico* means to predict the risk associated with individual drugs is a complex process for which the optimal implementation remains to be decided upon. In this article we will consider what level of mechanistic detail describing the interaction between drug and ion channel target needs to be included for *in silico* models to accurately predict risk and further, how much of this detail can be retrieved from protocols that are practical to implement in high throughout screens as part of preclinical development?

## Complexity of the Kv11.1/drug interaction

In many cases, a drug's interactions with its ion channel pharmacological target can be described as a simple bimolecular reaction according to the equation:

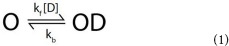



Where O represents the open ion channel, D is drug, and *k*_*f*_and *k*_*b*_ are the rates of association and dissociation respectively. A dissociation constant (*K*_*D*_), describing the affinity of binding can then be defined as the quotient of *k*_*b*_ from *k*_*f*_:

(2) $$\begin{array}{r} K_{D}=\frac{k_{b}}{k_{f}} \end{array}$$

In the scenario where this binding results in block of the ion channel current, the IC_50_, the drug concentration at which 50% of channels are blocked, approximates the *K*_*D*_. Whilst Kv11.1 interaction with drugs does not follow this simple rule (Windley et al., 2016), it nevertheless provides a useful framework for discussion of drug binding to Kv11.1. A detailed consideration of the factors that contribute to the complexity of block of these channels is presented in the following sections.

### Drug binding kinetics

The inclusion of drug binding kinetics in *in silico* simulations has been demonstrated to significantly alter predictions of cardiac action potential prolongation (Di Veroli et al., 2014; Lee et al., 2016). Specifically, drugs of equivalent affinity for Kv11.1 demonstrate varying degrees of action potential prolongation (Figures 1B–D). In some experiments, up to 4-fold increased difference in prolongation can be observed when comparing equipotent drugs with fast kinetics (τ_on_ = 0.1 s) to those with slow kinetics (τ_on_ = 100 s) (Di Veroli et al., 2014), which is within the range of time constants for drug binding observed for known drugs (Windley et al., 2017). Moreover, this differential prolongation is accentuated at different pacing frequencies; fast drugs cause greater prolongation at lower pacing frequencies while the opposite is true for slow drugs. At a pacing cycle length of 1,000 ms this leads to a difference in prolongation of APD_90_ of 52 ms when these parameters are incorporated into *in silico* simulations (Figure 1C; Lee et al., 2016).

These rate dependent effects therefore contribute to one of the most commonly measured indicators of proarrhythmic propensity—reverse rate dependence (RRD)—where an inverse relationship exists between action potential prolongation and depolarization frequency (Hondeghem et al., 2001a,b). The implied mechanism of this is that drugs with different kinetics of binding reach different levels of steady state block as a function of the relative rates of drug binding, unbinding and cycle length. Specifically, for the drugs shown in Figures 1B–D, this manifests as a maximal 30% block of Kv11.1 achieved with application of slow drugs at an IC_50_ dose during 1 Hz pacing, compared to 50% block for fast drugs under the same conditions (Lee et al., 2016). While it is known that other factors including genetic background and environmental factors including adrenergic upregulation of I_Ks_ (Sanguinetti et al., 1991; Bosch et al., 2002; Bányász et al., 2009) contribute to RRD, it is clear that the kinetics of the drug/channel interaction are also central to this established measure of proarrhythmia.

**Figure 2:**
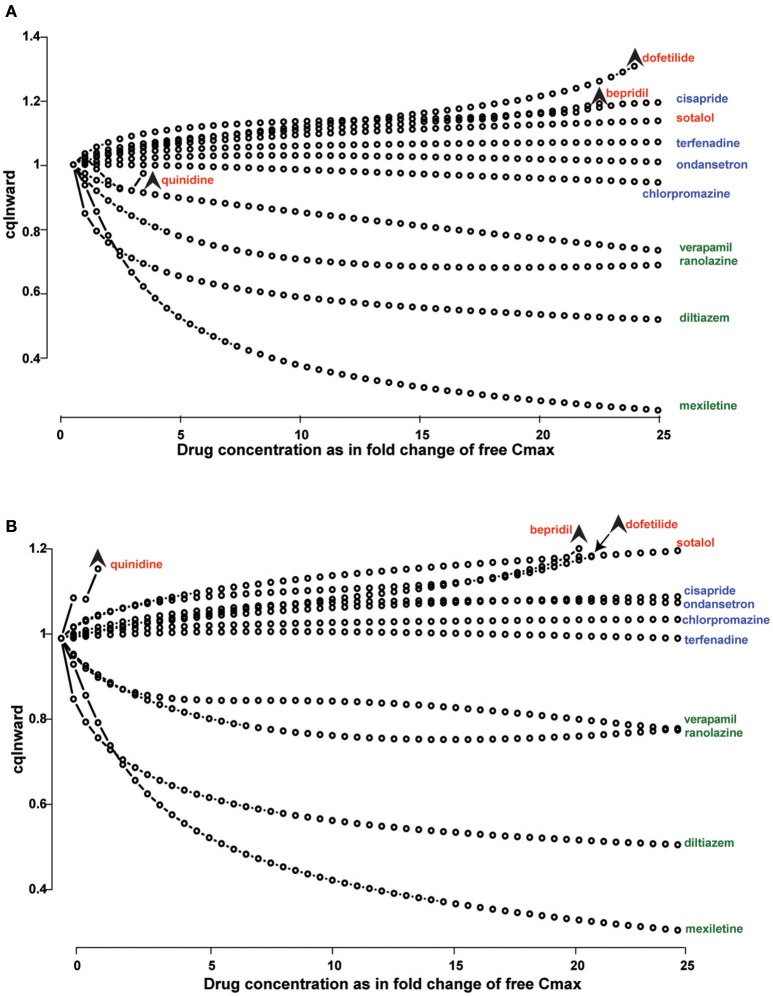
Drug binding kinetics improves risk prediction. **(A,B)** Concentration-dependent cqInward for all CiPA (Comprehensive *in vitro* Proarrhythmia Assay) training compounds. (High risk drugs are labeled in red, intermediate risk in blue and low risk in green) x-axis is the ratio between the simulated concentration and free *C*max; y-axis is the cqInward metric. Stars indicate the threshold dose, which is the highest dose that did not elicit an early afterdepolarization (EAD). The metric cqInward is the net inward current for each drug and is calculated as (INaL_AUC_drug/INaL_AUC_control+ICaL_AUC_drug/ICaL_AUC_control)/2, where AUC is the integrated area under the curve of the late sodium (INaL) and L-type calcium (ICaL) current traces during steady-state action potential simulation with (_drug) or without (_control) drugs. **(A)** Simulations are performed using instantaneous block of Kv11.1 base on dose response curves. **(B)** Simulations are performed using a dynamic model of Kv11.1-drug binding with incremental improvement in arrhythmia risk stratification. Reproduced from Li et al. (2017).

Another characteristic of drug interaction with Kv11.1 that is at least partially underpinned by the kinetics of binding and unbinding is that of “trapping” (Carmeliet, 1992; Yang et al., 1995; Mitcheson et al., 2000b; Perry et al., 2004; Stork et al., 2007). For some drugs, this phenomenon is due to true drug trapping. In these cases the drug molecule remains within the channel pore, sterically prevented from diffusing out as a result of closing of the cytoplasmic gate when the channel deactivates (Mitcheson et al., 2000b; Stork et al., 2007). Other compounds however, are more likely to display “virtual trapping,” where drug unbinding is significantly slower than the rate of channel deactivation (Perry et al., 2004). In these cases, depending on the voltage protocol used, the drug will appear to be “virtually trapped” if the interpulse time is insufficient for complete drug dissociation. (Lee et al., 2016; Windley et al., 2017). However, the extent to which the degree and type of trapping can be measured *in vitro* using simple voltage protocols is limited. For example, in the step depolarization protocol used by Windley et al. (2017) and Li et al. (2017), the degree of trapping is estimated with a fixed 15 s interpulse interval. The limitations of this approach are twofold. First, it is not possible to distinguish between true trapping and virtually trapped drugs. In practical terms, *in silico* simulation has demonstrated that true trapping results in significantly greater APD_90_ prolongation and greater pro-arrhythmic risk, Di Veroli et al. (2014) and therefore it is important to distinguish between the two. Second, any virtually trapped drug that dissociates quicker than 15 s will be described as non-trapped. Even if there was significant residual block evident at 5 or 10 s, the protocol cannot test this. In the context of cardiac cycle, where a typical diastolic interval might be on the order of 600 ms, this might be a significant shortcoming. Even so, Li et al. have shown that including an approximation of trapping and kinetics based on this protocol in their *in silico* models is useful in improving proarrhythmic prediction. For example, discrimination between drugs with low, medium and high risk of proarrhythmia using the metric ability of the “cqInward” (which represents the net inward current during the cardiac action potential) is incrementally improved when descriptions of trapping and kinetics are included in simulation as compared to simple IC_50_ measures of drug potency (Li et al., 2017; Figure 2). This is therefore clearly an important factor to consider, even if it is described with some degree of simplicity.

In summary, the kinetics of binding and unbinding of drugs to Kv11.1 play an important role in accurate prediction of prolongation of repolarization and proarrhythmic propensity. The addition of greater complexity, such as variability in ionic channel densities/function seen in different layers of the ventricular myocardium (Saiz et al., 2011) or congenital LQTS mutants (Romero et al., 2014), will further compound these effects. Consequently, in pursuit of a robust and comprehensive risk prediction assay, detailed understanding of these baseline measures of drug binding kinetics are likely an important component for *in silico* risk prediction and inaccurate estimation will likely lead to compounding errors as we continue to build and refine prediction models.

### State dependent binding

The vast majority of drugs which target Kv11.1 require channel opening in order to gain access to the receptor site within the inner cavity of the channel pore (Kiehn et al., 1996; Walker et al., 1999; Vandenberg et al., 2012). However, a drug's affinity can be relatively greater for either the open state (K_o_) or inactivated state (K_i_) resulting in a state preference in drug binding. To date, there are no studies that have been able to demonstrate drug binding in the closed state. Several studies have demonstrated that some, but not all, drugs with high affinity binding to Kv11.1 show preferential binding to the inactivated state (Suessbrich et al., 1997; Ficker et al., 1998; Numaguchi et al., 2000; Perrin et al., 2008a; Du et al., 2014). These studies show that Kv11.1 binding potency in certain high affinity drugs is reduced by using inactivation attenuated mutants, such as N588K or S631A (Perrin et al., 2008a; Du et al., 2014), or the inactivation deficient mutant S620T (Suessbrich et al., 1997; Ficker et al., 1998; Perrin et al., 2008a; Wu et al., 2015). The inference being that these drugs favor binding to the inactivated state, hence channel mutants which reduced inactivation are less likely to bind the drug in question.

However, it is important to note that it is not always the case that there is a direct correlation between the extent of inactivation and the affinity of drug binding, even for state-dependent blockers. Electrophysiological studies using concatenated Kv11.1 tetramers containing variable number of inactivation deficient subunits have demonstrated changes in drug binding affinity that occur independent of inactivation (Chen et al., 2002; Wu et al., 2015). The major molecular determinants for drug binding to Kv11.1 are two aromatic residues Y652 and F56 in the S6 helix (Mitcheson et al., 2000a; Chen et al., 2002; Wu et al., 2015). In addition to these two S6 aromatics, Thr623, Ser624, and Val625 at the base of the selectivity filter, and Phe557 on the S5 helix also contribute to drug binding at least for some drugs (Mitcheson et al., 2000a,b; Saxena et al., 2016). It is likely that conformational changes that accompany inactivation, but that are not strictly necessary for the open to inactivated state transition, alter the arrangement of these residues within the pore cavity to allow for additional close molecular interactions that result in preferential binding to the inactivated state (Durdagi et al., 2012). This is supported by evidence from molecular dynamics simulations, albeit those limited to using homology models of Kv11.1, which demonstrate these conformational changes (Durdagi et al., 2012).

Of course, there are also non-state dependent drugs whose potencies are not affected by inactivation deficient mutants. These drugs include: quinidine, erythromycin, perhexiline (Perrin et al., 2008a), and clozapine (Hill et al., 2014). Moreover, studies have suggested that some compounds may also have an open state preference with minimal binding to the inactivated state (Kamiya et al., 2001; Park et al., 2002; Su et al., 2004). However, the contention with these studies is that rather than using mutagenesis to manipulate state occupancy, they utilize complex non-standardized voltage protocols to demonstrate state preference since an open deficient Kv11.1 mutant is not useful due to an absence of current. It is likely that the recent advent of high resolution structures of Kv11.1 (Wang and MacKinnon, 2017) and the potential this presents to generate more structures of drugs interacting with inactivation deficient Kv11.1 channels, will allow more accurate molecular dynamic simulations to probe these questions around state-dependent drug binding in more detail.

**Figure 3:**
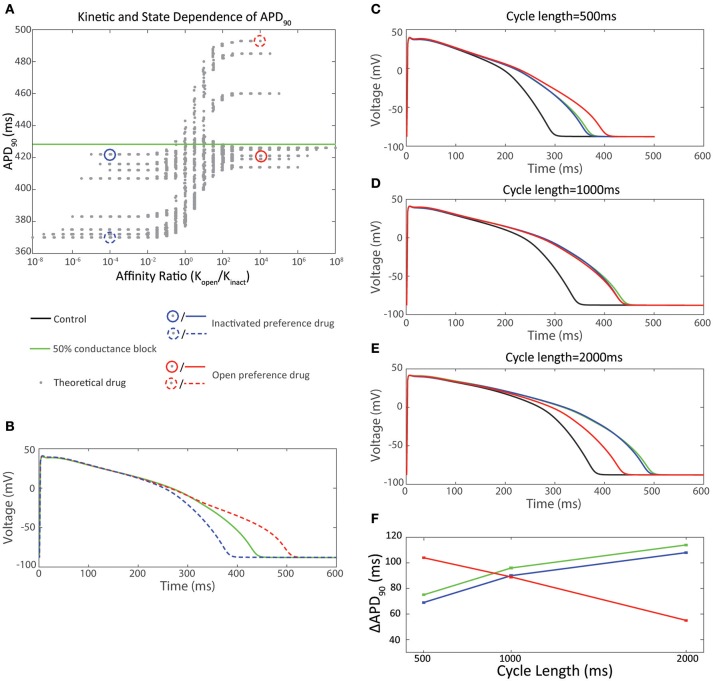
Effect of state preference on cardiac action potential prolongation. **(A)** 2 drugs with equal but opposite ratio of affinity for the open vs. inactivated state were selected from the dataset in **(A)**. The inactivated state preference drug (*K*_o_ /*K*_i_ = 10^−4^) is shown in blue-dash. The open state preference drug (*K*_o_ /*K*_i_ = 10^4^) is shown in red-dash. **(B)** Ventricular action potentials corresponding to the two drugs in **(A)** in comparison to IK_r_50. **(C–F)** Effect of pacing cycle length on cardiac action potential prolongation. 2 drugs with equal and opposite ratio of affinity for the open vs inactivated state and equal APD_90_ at a pacing cycle length of 1,000 ms were selected from **(A)**. The inactivated state preference drug (*K*_o_ /*K*_i_ = 10^−4^) is shown in blue-solid. The open state preference drug (*K*_o_ / *K*_i_ = 10^4^) is shown in red-solid. **(C)** Action potentials at 500 ms pacing interval. **(D)** Action potentials at 1,000 ms pacing interval. **(E)** Action potentials at 2,000 ms pacing interval. **(F)** Pacing cycle dependence of ΔAPD_90_

How important then is the consideration of state dependent binding for *in silico* prediction of arrhythmic risk? The data in Figures 3A,B shows that an IC_50_ dose of two drugs with opposite state preferences differ in the degree of observed APD prolongation by 56 ms—clearly a significant amount in predicting their proarrhythmic potential. However, this relationship also needs to be considered through the prism of the limitation that the measured IC_50_ is itself influenced by the state preference and how this manifests as a function of the voltage protocol used to measure the potency. Current safety guidelines mandate equilibrium testing of drugs to estimate potency to estimate arrhythmic risk (S7B, 2005). However, measures of drug potency vary between voltage protocols for some drugs but not for others (Kirsch et al., 2004; Yao et al., 2005; Milnes et al., 2010). These differences, which can be an order of magnitude in disparity, are in part due to using voltage protocols which favor occupancy of either the open or inactivated state, so favoring drug binding to that state (Milnes et al., 2010). Therefore, how can one measure state preference for incorporation into *in silico* simulations? The processes of channel opening and inactivation occur over overlapping voltage ranges, so it is almost impossible to tease out the relative affinities for the two states from a single, relatively simple voltage protocol. One potential approach to this might be to examine multiple protocols, that each sample the state occupancy of open vs. inactive differently, and attempt to infer information about state preference from the differences in IC_50_s measured using each. However, this is a relatively complex task that may not be amenable to high throughput screens.

### Temperature dependence

The temperature dependence of potency of Kv11.1 block is a phenomenon that has been described in the literature for many drugs (Guo et al., 2005; Yao et al., 2005; Alexandrou et al., 2006; Hill et al., 2007). For instance, Guo et al. (2005) and Alexandrou et al. (2006) demonstrate an increase in potency with respect to increasing temperature from 22 to 37°C for fluoroquinolone antibiotics (erythromycin and moxifloxacin respectively), although not to the same magnitude. In contrast, other drugs, including loratadine and bepridil, exhibit reduced potency at physiological temperatures (Kirsch et al., 2004). Larger scale studies have also established that a range of different drugs have a variable degree of Kv11.1 blockade when examined at ambient temperature compared to physiological temperatures (Kirsch et al., 2004; Yao et al., 2005). Moreover, differences in temperature sensitivity can be accentuated by different voltage protocols (Kirsch et al., 2004).

In relation to gathering data to constrain *in silico* models, this problem is further complicated by recent studies by Windley et al. (Windley et al., 2016), that revealed some mechanistic insights into the temperature dependence of drug binding to Kv11.1. Using a direct measurement of kinetics at 0 mV rather than a pulsed voltage protocol, Windley et al. demonstrated that for cisapride, increasing temperature from 22 to 37°C did not affect affinity of binding, but significantly altered kinetics. *In silico* the temperature dependence of binding and unbinding kinetics could not be described by a simple bi-molecular interaction, but required inclusion of an “encounter-complex”; a conducting intermediary state between unblocked and blocked channels states. This change in kinetics could also be used to potentially explain apparent differences in drug potency when using varying pulsed voltage protocols as discussed in the following section For example: at 20 nM comparing 22–37°C, Windley et al. observed a 5.5-fold increase in cisapride binding rates to Kv11.1. Based on the analysis in Lee et al. (2016), this could account for a ~20 ms increase in drug-induced APD_90_ prolongation, despite no change in affinity. In addition to these effects of temperature on drug binding, the gating of the Kv11.1 channel itself is sensitive to temperature. Specifically, at 37°C there are increases in channel conductance, a hyperpolarizing shift in activation and a depolarizing shift in inactivation (Vandenberg et al., 2006), resulting in an overall increase in in open state that will also influence the measured potency of drugs that display state dependent binding (see Section State Dependent Binding).

The implications of these studies are that for some drugs, measures of potency and kinetics made at ambient temperatures may not be useful in constraining *in silico* models used to predict proarrhythmia at physiological temperatures (Windley et al., 2016). This is potentially a concern for large scale, high-throughput drug screening as many of the current generations of automated patch clamp platforms are limited to recording at ambient temperature (Fermini et al., 2016). However, efforts are currently underway, through the CiPA *in silico* Working Group and High Throughout stream to establish the practical importance and consequences of this issue.

## Measuring and modeling drug binding to Kv11.1

The complexity of Kv11.1-drug interactions therefore has clear implications for how the field should approach both measuring of these phenomena *in vitro*, as well as how we describe them using *in silico* models that can be used for risk prediction. Current guidelines stipulate the *I*_Kr_ current should be assayed but do not specify voltage protocols, or details, such as temperature or cellular expression systems. As a result there is a lack of standardization in how block of *I*_Kr_ current is measured (Fermini et al., 2016). Redfern et al. (2003) proposed a 30-fold safety margin between the measured IC_50_ of a drug and its maximum unbound plasma concentration (C_max_) to distinguish between safe and unsafe drugs. However, many studies have shown variance in drug potency dependent as a function of temperature and voltage protocol (Kirsch et al., 2004; Yao et al., 2005; Milnes et al., 2010) and this safety margin becomes unreliable if a true IC_50_ value cannot be agreed upon. For example, the reported IC_50_ values for cisapride, span a 60-fold range (Potet et al., 2001; Rezazadeh et al., 2004). One approach therefore is to use *in silico* modeling to “fine-tune” *in vitro* experimental protocols to more closely mimic *in vivo* conditions (Ellinwood et al., 2017). However, even if a standardized protocol could be agreed upon, such as using a physiological cardiac action potential to reproduce the state transitions of Kv11.1 that are seen during the cardiac cycle, this would not take into account the impact of variations in heart rate or action potential prolongation which are paramount to the highly dynamic binding kinetics of the drug/Kv11.1 interaction. For example the data in Figures 3C–F shows two drugs with equal and opposite gating state preference. At 1,000 ms pacing cycle length the APD_90_ differs by 1 ms. However, at 500 ms pacing cycle length the open state preference drug prolongs the APD_90_ by 35 ms *more* than the inactivated state preference drug; while at 2,000 ms pacing cycle length the open state preference drug prolongs the APD_90_ by 53 ms *less* than the inactivated state preference drug. (Figures 3C–F) Moreover, these standardized conditions also lack the ability to predict variations in physiological conditions, such as hyper/hypo- kalaemia (Wang et al., 1997) or low pH (and the consequent changes in protonation of drug compounds) (Moreno et al., 2011; Wang et al., 2016), all of which are known to affect the state-dependence of drug binding. An alternative therefore, is to use non-physiological voltage protocols to accurately constrain *in silico* models of drug binding (Hill et al., 2014; Beattie et al., 2017) that can then be used *in silico* to evaluate a limitless range of physiological conditions.

**Figure 4:**
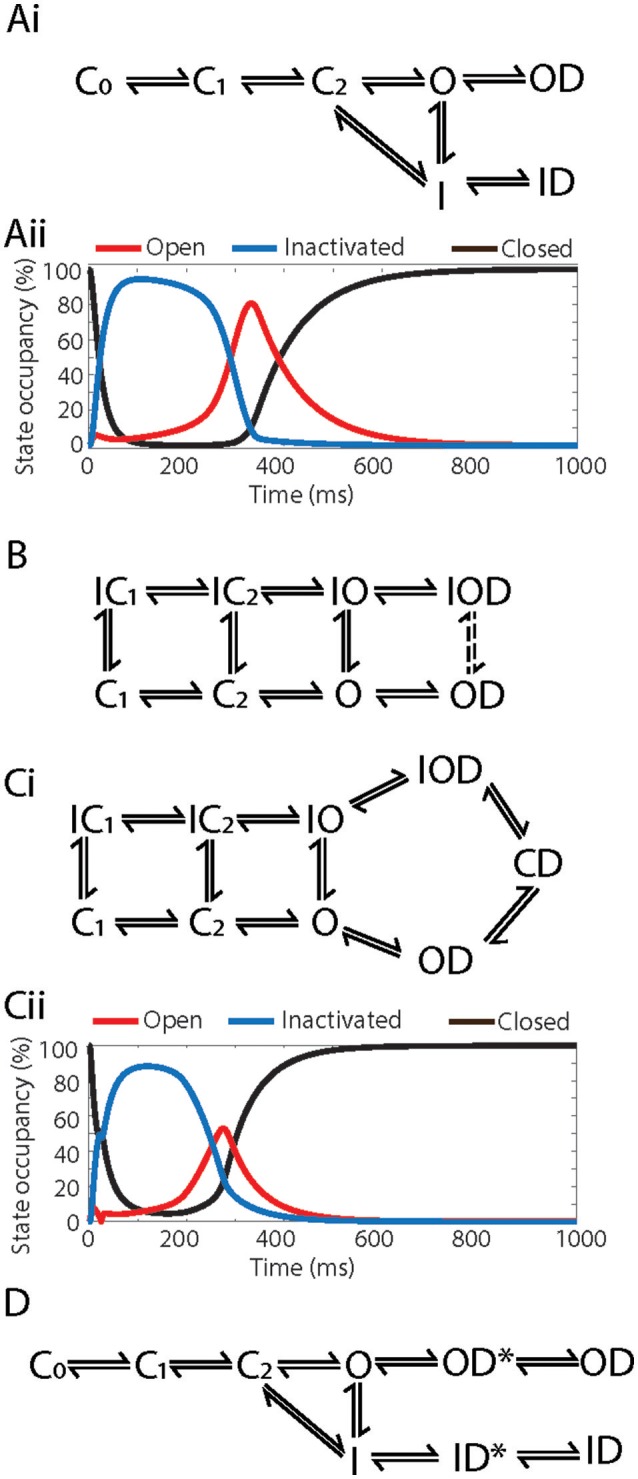
Example Markov models of drug binding to Kv11.1. **(Ai)** Kinetic and gating-state dependent model a*dapted from* Lee et al. (2016). **(Aii)** State occupancies of the combined closed (C-black), open (O-red) and inactivated (I-blue) states using the Markov model in **(Ai)**, simulated in an O'Hara Rudy action potential at 1 Hz. **(B)** Kinetic and gating-state dependent model a*dapted from* Di Veroli et al. (2013). **(Ci)** Drug trapping model *adapted from* Li et al. (2017). **(Cii)** State occupancies of the combined closed (C-black), open (O-red) and inactivated (I-blue) states using the Markov model in **(Ci)**, simulated in an O'Hara Rudy action potential at 1 Hz. **(D)** Temperature dependent model *adapted from* Windley et al. (2016). C_0_, C_1_, C_2_, Closed states; IC_1_, IC_2_, Inactivated-closed states; I, IO, Inactivated states; O, Open state; ID, IOD, Drug bound-inactivated states; OD, Drug bound-open state; CD, Drug trapped state.

This approach however brings with it a new set of challenges. There exists a wide variety of models of Kv11.1/drug interaction in the literature, each with different structures and each constrained by different *in vitro* datasets. Furthermore, they differ substantially in their ability to describe the key features of Kv11.1/drug binding dynamics discussed above, such as kinetics and state dependence (Figures 4A,B; Di Veroli et al., 2013; Hill et al., 2014), drug trapping (Figure 4C; Li et al., 2017) and temperature dependence (Figure 4D; Windley et al., 2016). While each of these models represents a good description of drug binding under certain conditions, they differ significantly in their predicted state occupancies over any given voltage protocol (Figures 4Aii,Cii), so will result in a difference in state-dependent drug binding. As yet, no Markov model provides a universal solution that we can be sure would be useful for prediction of proarrhythmic risk. As a result, further complexity may need to be added, or the existing models constrained with new *in vitro* data, to improve the model's predictive accuracy (Fermini et al., 2016). The issue of what is the optimum approach to measuring and modeling drug binding to Kv11.1 is therefore an open question and the optimum balance between how much and what type of data is required to constrain *in silico* models and what is practical to do in the context of high throughput data acquisition is yet to be determined.

## Multichannel pharmacology

**Figure 5:**
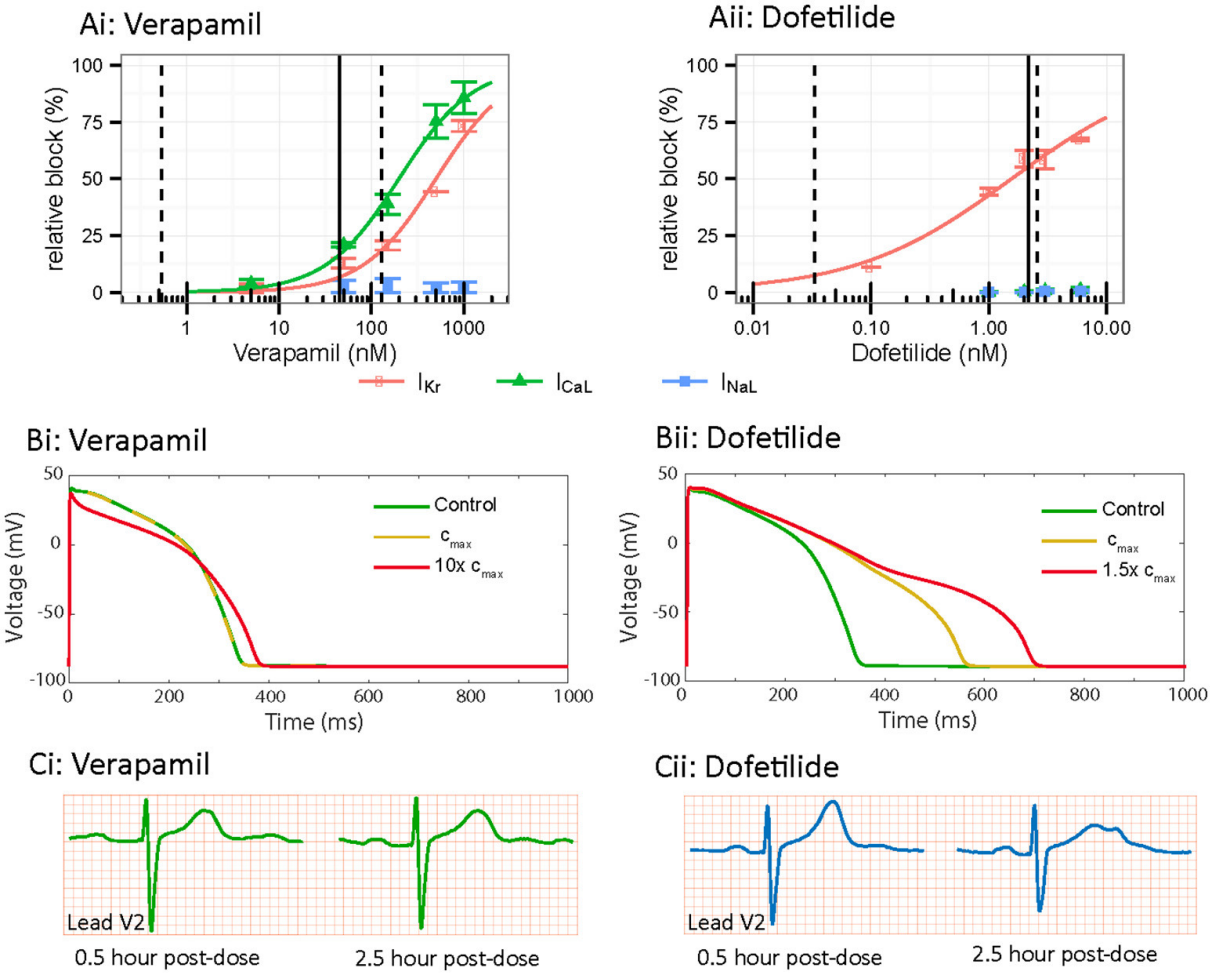
*In vitro, in silico* and *in vivo* comparison of multi-channel pharmacology. **(A)** *In vitro* drug block of Kv11.1 (I_Kr_-red), Cav1.2 (I_CaL_-green) and Nav1.5-Late (I_NaL_-blue) for (i) verapamil and (ii) dofetilide. Adapted from Vicente et al. (2015). **(B)** *In silico* cardiac action potential prolongation and morphology based on *in vitro* data from Vicente et al. (2015), simulated using the O'Hara Rudy ventricular action potential. Control is shown in green, C_max_ dose in yellow and super-C_max_ dose in red. Verapamil demonstrates less action potential prolongation and triangulation compared to Dofetilide. **(C)** *In vivo* ECG data demonstrating lead V2 in a patient at 0.5 and 2.5 h post injection of 120 mg verapamil or 500 ug dofetilide. Verapamil demonstrates no change in prolongation or T-wave morphology while Dofetilide shows marked changes in prolongation and T-wave notching between the 2 time points. Reproduced from Vicente et al. (2015).

The final piece of the puzzle that needs to be considered in developing ventricular cell simulations for *in silico* risk prediction is the role of multichannel pharmacology, and how this contributes to characteristics of the cellular action potential. Whilst Kv11.1 blockade is certainly critical to understanding aLQTS and drug induced TdP, it is not the sole determinant of arrhythmogenesis since drugs that block Kv11.1 can often also block other cardiac ion channels (Bril et al., 1996; Aiba et al., 2005; Wu et al., 2008; Vicente et al., 2015) to suppress or promote arrhythmogenesis (Fermini et al., 2016). An evaluation of the potency of a panel of 30 drugs against the seven major currents that contribute to repolarization demonstrated that the primary pharmacological targets that determine proarrhythmia were I_Kr_ (Kv11.1), I_CaL_ (Cav 1.2), and I_NaL_ (Nav1.5-late). Furthermore, drugs with high TdP risk tended toward unopposed Kv11.1 block, while drugs with low TdP risk had similar or higher potency for the inward currents (I_CaL_ and I_NaL_) in conjunction with Kv11.1 block (Crumb et al., 2016). These multichannel pharmacological profiles are reflected in the morphology of the AP waveform (and hence the surface ECG). The AP waveform is formed through summed contribution of all the individual ionic currents in the cardiac myocytes. As a result, varied drug block of different ionic currents will result in a spectrum of AP morphologies and durations, which is idiosyncratic to individual drugs that manifests *in vivo* as differences in QT duration as well as T wave morphology (Figures 5Ci–Cii; Vicente et al., 2015). Critically for *in silico* risk prediction, this “AP morphology signature” is in turn linked to the drug's pro-arrhythmic potential and potentially can be used to predict TdP.

In this regard, drug induced morphological changes in the cardiac AP have been shown to correlate with risk of TdP (Hondeghem et al., 2001a). Specifically, this study suggested the presence of AP “triangulation” (slow repolarization, without a distinct plateau or rapid repolarization phase) was a marker of risk of drug induced TdP. Several single drug studies exemplify this point and support the link with multichannel pharmacology. Drugs that block Kv11.1 without significant inward current block, such as Sotalol (Milberg et al., 2004) and dofetilide (Osadchii, 2012; Figure 5Aii), produce AP triangulation in addition to prolongation, (Figure 5Bii) and are considered high TdP risk drugs. Conversely, Verapamil, a potent blocker of Cav1.2 as well as Kv11.1 (Figure 5Ai), does not manifest in triangulation or prolongation of the AP (Figure 5Bi) and is considered a low TdP-risk drug (Aiba et al., 2005). Similarly, other drugs with multichannel pharmacological profiles, such as ranolazine (Jia et al., 2011) and tolterodine (Martin et al., 2006) demonstrate dose dependent AP prolongation without AP triangulation and are also considered low risk. It is clear therefore, that multichannel pharmacology, and its manifestation in morphology of the AP, is an important detail that must be considered for risk prediction.

*In silico* modeling again provides an ideal solution to integrating pharmacological data from multiple cardiac ion channels. Indeed, recent studies by Li et al. (2017) and Dutta et al. (2017) have shown the value of this approach and demonstrated that incorporating Cav1.2 and Nav1.5-late block into action potential simulations improves arrhythmia risk prediction (Yang et al., 2016; Li et al., 2017). However, in a similar vein to that discussed above for models of the Kv11.1/drug interaction, there are several models of the ventricular cardiac action potential that have been proposed in the literature including the ten Tusscher 2006 (TT06) (Ten Tusscher and Panfilov, 2006), Grandi-Bers 2010 (GB10) (Grandi et al., 2010), and O'hara Rudy 2011 (ORD11) models (O'Hara et al., 2011). Population based studies using these cell models have allowed interrogation of how the variation in repolarization reserve that occurs as a result of differential expression of ion channels between individuals can influence predicted drug effects as well as develop our understanding of multichannel pharmacology (Sobie, 2009; Lancaster and Sobie, 2016). However, each of the models is considerably different in relation to the conductance levels of individual cardiac ion channels. As a result, predictions around APD prolongation and emergence of proarrhythmic markers that each of the models make in response to drug block are significantly different (Mirams et al., 2014) and do not reproduce *in vivo* data (Britton et al., 2017). For example, Mann et al showed that 50% inhibition of Kv11.1 caused 113, 22, and 34 ms prolongation of APD_90_ for ORD11, TT06 and GB10 respectively (Mann et al., 2016). This issue is being considered by the field and recent efforts have focused on refining cell models by rescaling their ionic conductances using either patient data from subjects with various subtypes of the long QT syndrome (Mann et al., 2016) or published drug data (Britton et al., 2013; Dutta et al., 2016). Even so, significant disparity still exists between the “optimized” versions of the cell models, meaning the differences in predicted risk that result from using different models are likely to outweigh, or at least match, the differences associated with descriptions of the drug/channel interaction. It may also prove to be true that similar mechanistic descriptors that are becoming routine for drug binding to Kv11.1, such as kinetics and state dependence, also need to be incorporated for other cardiac ion channels for optimum risk prediction. However, the benefit of this relative to the cost of acquiring the data may preclude such an approach. What is clear, is that each of these facets of *in silico* risk prediction—the Markov descriptions of drug/channel interaction *as well as* the model of the ventricular cell in which they are incorporated, should each be considered as a priority for the field.

## Conclusion

Understanding the intricacies of the Kv11.1/drug interaction and optimizing our approaches to measuring and modeling these characteristics is critical to developing better preclinical *in silico* risk prediction. In doing this it is important to remember that all models are simplifications. Therefore, the challenge is to determine how much information needs to be included to make them useful rather than how much information is needed to make them accurate for every drug scenario, which would potentially necessitate the collection of very large amounts of data that may be redundant for many drugs. Given the potential significance of factors, such as drug binding kinetics, temperature dependence, state dependence and multichannel pharmacology discussed above, it seems clear that these factors need to be included at some level in models of drug binding. The major challenge faced by the field in the short term is determining what level of detail is necessary, and balancing this against the practicalities of data acquisition in high throughout screens.

## Author contributions

WL, MW, JV, and AH all contributed to planning, writing and editing of the manuscript and figures contained herein.

### Conflict of interest statement

The authors declare that the research was conducted in the absence of any commercial or financial relationships that could be construed as a potential conflict of interest. The reviewer SM and handling Editor declared their shared affiliation.
